# Genomic characterization of *Mycobacterium tuberculosis* lineage 7 and a proposed name: ‘Aethiops vetus’

**DOI:** 10.1099/mgen.0.000063

**Published:** 2016-06-24

**Authors:** Hanna Nebenzahl-Guimaraes, Solomon A Yimer, Carol Holm-Hansen, Jessica de Beer, Roland Brosch, Dick van Soolingen

**Affiliations:** ^1^​ Tuberculosis Reference Laboratory, National Institute for Public Health and the Environment (RIVM), Bilthoven, The Netherlands; ^2^​ Department of Microbiology, Genome Dynamics and Microbial Pathogenesis Group, Oslo University Hospital, Oslo, Norway; ^3^​ Infection Control and Environmental Health, Norwegian Institute of Public Health, Oslo, Norway; ^4^​ Unit for Integrated Mycobacterial Pathogenomics, Institut Pasteur, Paris, France; ^5^​ Department of Medical Microbiology, Radboud University Medical Centre, Nijmegen, The Netherlands

**Keywords:** Tuberculosis, phylogenetic lineage, Ethiopia

## Abstract

Lineage 7 of the *Mycobacterium tuberculosis* complex has recently been identified among strains originating from Ethiopia. Using different DNA typing techniques, this study provides additional information on the genetic heterogeneity of five lineage 7 strains collected in the Amhara Region of Ethiopia. It also confirms the phylogenetic positioning of these strains between the ancient lineage 1 and TbD1-deleted, modern lineages 2, 3 and 4 of *Mycobacterium tuberculosis*. Four newly identified large sequence polymorphisms characteristic of the Amhara Region lineage 7 strains are described. While lineage 7 strains have been previously identified in the Woldiya area, we show that lineage 7 strains circulate in other parts of the Amhara Region and also among foreign-born individuals from Eritrea and Somalia in The Netherlands. For ease of documenting future identification of these strains in other geographical locations and recognizing the place of origin, we propose to assign lineage 7 strains the lineage name *‘*Aethiops vetus*’*.

## Data Summary

1. Reads and assembled scaffolds have been deposited in the European Nucleotide Archive (study accession number PRJEB8432; http://www.ebi.ac.uk/ena/data/view/PRJEB8432).

## Impact Statement

This article communicates novel insights gained from genomic data on the new lineage 7 of *Mycobacterium tuberculosis* (Mtb), namely to describe its positioning relative to other Mtb strains and the specific variations (SNPs, insertions and deletions) that distinguish this lineage from other lineages and (sub) species in the complex. This novel information will contribute towards the burgeoning awareness of this lineage that is of considerable evolutionary interest due to its intermediary positioning between ancient and modern lineages. Further investigation of differences in virulence and immunogenicity among Mtb major lineages, including lineage 7, is warranted to elucidate the pathogenesis and clinical consequences of TB disease. For ease of documenting the global epidemiology and anticipating future trends, we propose to name the lineage ‘Aethiops vetus’, which reflects the ancient origin of these strains in the Horn of Africa.

## Introduction

A new lineage within the *Mycobacterium tuberculosis* complex (MTBC) has recently been identified among strains originating from Ethiopia (Firdessa *et al.,* 2013; Blouin *et al.*, 2012). This lineage, named lineage 7, is of considerable interest regarding evolutionary research as it represents a phylogenetic branch intermediate between the ancient and modern lineages of *Mycobacterium tuberculosis (*Mtb). Further characterization of this lineage may provide insight as to where, when and how Mtb evolved, as well as what influence human hosts had on the development of these bacteria. This is important for a better understanding of Mtb adaptation to the human host, and the highly developed ability of the bacterium to evade the immune system and spread extensively in the human population. Characterization is thus essential to elucidate pathogenicity, which underlies vaccine development, to document the global epidemiology of tuberculosis (TB), and to anticipate future trends in the spread of the disease.

In this study we explore the positioning of this new lineage relative to other Mtb strains through different DNA typing techniques, and identify the variations, large sequence polymorphisms (LSPs) and smaller deletions, which distinguish the lineage from other groupings in the complex. In addition, we describe lineage 7 strains detected in The Netherlands where the majority of the patients are foreign-born. Lastly, to document the place where lineage 7 strains have been identified and possibly originate, we suggest naming these strains ‘Aethiops vetus’.

## Methods

### Isolate details.

Six MTBC isolates were cultivated from sputum samples collected from newly diagnosed pulmonary TB (PTB) patients attending selected health centres and hospitals in various districts of Amhara Region, Ethiopia. The isolates were part of 237 Mtb isolates collected between 2008 and 2010 for the TB Rapid Test project at the Norwegian Institute of Public Health (NIPH). Bacteriological and molecular characterization of the strains was performed in Ethiopia, Norway and The Netherlands. Prior to sputum collection, socio-demographic and clinical data were obtained using a structured questionnaire. HIV testing was also performed on blood samples obtained from study participants following the routine procedure in Ethiopia.

### Sputum smear microscopy, culture and drug susceptibility testing.

Three consecutive sputum samples, spot–morning–spot (Rao, 2009), were provided by study participants. Smear microscopy using Ziehl-Neelsen staining was performed to identify acid-fast bacilli. Pooled sputum samples were transported to the Armauer Hansen Research Institute (AHRI) in Addis Abeba, Ethiopia for culture. Sputum culture was performed using the conventional Löwenstein–Jensen (LJ) culture media method (Christie & Callihan, 1995). Drug susceptibility testing (DST) was conducted at the Ethiopian Health and Nutrition Research Institute (ENHRI), the national TB reference laboratory in Addis Abeba, and at AHRI. DST was performed by the proportional absolute concentration technique (Van Klingeren *et al.*, 2007) and the BBL MGIT Mycobacterium Growth Indicator Tube (BD) method following the manufacturer’s instructions (Siddiqi & Rüsch-Gerdes, 2006).

### DNA extraction and spoligotyping.

Deoxyribonucleic acid (DNA) was extracted from Mtb isolates according to standard procedure (Zwadyk *et al.*, 1994). Species identification by polymerase chain reaction (PCR) targeting the RD9 (region of difference 9) was performed as previously described (Parsons *et al.*, 2002). Genomic regions of difference (RDs) denote large deletions spanning several genes and are used for the differentiation of members of the MTBC (Warren *et al.*, 2006).

DNA of the Mtb isolates was subjected to spoligotyping at AHRI according to Kamerbeek *et al.* (1997), and the results and DNA aliquots were sent to the Tuberculosis Reference Laboratory at the National Institute for Public Health and the Environment (RIVM) in The Netherlands for quality control. The results indicated that the spoligotyping performed at AHRI was in agreement with the findings at RIVM (Yimer *et al.*, 2013). The International Spoligotype Database (SpolDB4, 2011) was used to assign the spoligotyping patterns to major genetic lineages and sub-lineages (Brudey *et al.*, 2006). New spoligo patterns that were not described by SpolDB4 were assigned to Mtb families by SpolDB3 and a randomly initialized model (Run SPOTCLUST, 2011).

### MIRU-VNTR typing.

Mycobacterial interspersed repetitive units (MIRU) variable number of tandem repeats (VNTR) typing was performed at NIPH in Oslo, Norway to improve the discrimination of Mtb isolates and to compare the results with the extended collection of VNTR profiles of strains collected within The Netherlands. VNTR is based on the identification of 24 of the most polymorphic microsatellite sequences (55–72 nucleotides in length) in the Mtb genome (Supply *et al.*, 2006). The findings are presented as a set of digits that indicate the number of tandem repeat sequences at each of the 24 loci (Alonso-Rodríguez *et al.*, 2008).

### Sequencing, assembly and variant calling.

Among the six isolates transferred to RIVM for whole-genome sequencing, one (Solo 80) could not be subcultured despite repeated attempts. DNA was thus extracted from five isolates using standard methods. Whole-genome sequencing was successfully performed by BaseClear BV (Leiden, The Netherlands) on an Illumina HiSeq 2500 instrument using reads of 50 bp in length in the paired-end modus. The average genome coverage was approximately 60-fold. The FASTQ sequence reads were generated using the Illumina Casava pipeline version 1.8.3. Initial quality assessment was based on data passing the Illumina Chastity filtering. Subsequently, reads containing adapters and/or PhiX control signals were removed using an in-house filtering protocol. The second quality assessment was based on the remaining reads using the FASTQC quality control tool version 0.10.0. The quality of the FASTQ sequences was enhanced by trimming off low-quality bases using the ‘Trim sequences’ option of the CLC Genomics Workbench version 6.5. The quality-filtered FASTQ sequences were assembled using Ray, and the resulting contigs were mapped to both *Mycobacterium tuberculosis* H37Rv (GenBank accession no. NC_000962.3) and *Mycobacterium africanum* GM041182 (GenBank accession no. FR878060.1). In parallel, single nucleotide polymorphisms (SNPs) and INDELS (insertions or deletions) were called using Breseq software (version 0.23) with a minimum depth of 15 x (Barrick *et al.*, 2009). SNPs with low-quality evidence (i.e. possible mixed read alignment) or within five base pairs (bp) of an INDEL were discarded. Reads and assembled scaffolds are deposited in the European Nucleotide Archive (study accession number PRJEB8432).

### Phylogenetic tree reconstruction and comparison of sequences.

A parsimony-based phylogeny (Phylip, version 3.695) of the MTBC was reconstructed using 78 613 concatenated variable positions (SNPs) that were found in at least one of 100 previously characterized RIVM strains of different lineages and the five strains from Ethiopia in relation to the outgroup strain *Mycobacterium canettii* CIPT 140070010 (strain STB-K; GenBank accession no. NC_019951.1). This strain was previously determined to be the most genomically distant relative to the core members of the MTBC (Supply *et al.*, 2013; Blouin *et al.*, 2014; Boritsch *et al.*, 2016). Two additional lineage 7 strains (Percy 256 and Percy 556) from an independent study were added to this tree (Blouin *et al.*, 2012). The Artemis Comparison Tool (ACT) (Carver *et al.*, 2008) was used to perform pairwise comparison between the different Ethiopian sequences, Mtb H37Rv and *M. africanum* GM041182 (Bentley *et al.*, 2012).

## Results

### Socio-demographic information

**Fig. 1.**
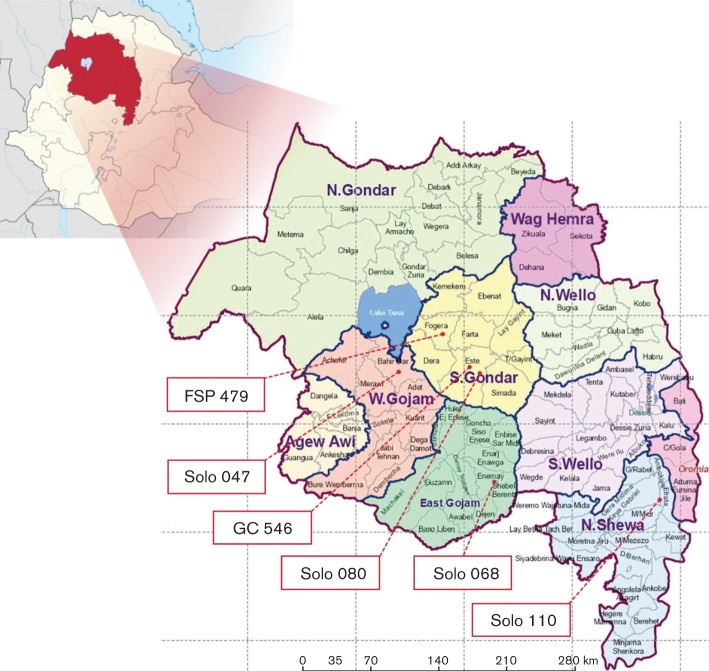
Map of Ethiopia and the Amhara Region locating specific sites where the six isolates were collected.

The five sequenced isolates were obtained from two female and three male study participants ranging in age from 15 to 50 years (mean age 32 years). All patients came from rural areas from five different districts of the Amhara Region (Fig. 1).

### Lineage 7 strains detected in The Netherlands

Isolates from three patients, two males and one female, originating from Somalia, Ethiopia and Eritrea, were previously collected in the Netherlands and identified as belonging to the new lineage 7. These three lineage 7 isolates were exceptional among the 3867 unique MIRU profiles stored in The Netherlands between 1993 and 2011. During this period, approximately 72 % of the patients were of foreign origin: 49 % from Africa, 32 % from Asia and 11 % from North or South America. Two of the lineage 7 strains were isolated in 2008, one in 2009, and all were sensitive to the five first-line antibiotics. Bacilli were isolated from positive sputum samples and the patients had only pulmonary manifestation. Contact investigation was performed for one case and one positive contact was identified (confirmed by thorax X-ray). All three were treated with the standard chemotherapeutic regimen and had a successful outcome.

### VNTR characterization of lineage 7 strains

The 24-locus VNTR typing patterns (the complete variation of patterns) of the eight lineage 7 strains (five isolated in Ethiopia and three in the Netherlands) are presented in Table 1. The similarity in VNTR patterns between the three lineage 7 strains found in The Netherlands with two of the present lineage 7 strains from Ethiopia ranged from 79.2 % to 95.8 % (average pairwise similarity of 86.1 %).

**Table 1.** 24-locus MIRU-VNTR typing patterns of eight lineage 7 strains

Sequence ID	Isolated in:	VNTR locus																							
		580	2996	802	960	1644	3192	424	577	2165	2401	3690	4156	2163b	1955	4052	154	2531	4348	2059	2687	3007	2347	2461	3171
68	The Netherlands	4	5	3	7	4	7	6	4	3	2	1	3	4	4	6	1	4	6	2	2	3	4	3	4
838		4	6	3	7	4	5	4	4	3	2	1	3	4	4	9	1	4	6	2	2	3	4	3	4
54		4	6	3	7	4	5	4	4	3	2	1	3	4	4	8	1	4	6	2	2	3	4	3	4
229	Ethiopia	4	6	3	7	4	6	2	4	3	2	1	3	4	4	8	1	4	6	2	2	3	3	3	4
230		4	6	3	7	4	5	4	4	3	2	1	3	4	4	8	1	4	6	2	2	3	3	3	4
232		8	6	3	7	4	5	4	4	3	2	1	3	4	4	9	1	4	6	2	2	3	4	3	4
233		4	6	2	7	4	5	4	4	3	2	1	3	4	4	2	1	4	6	2	2	1	3	3	4
234		4	6	3	7	4	5	4	4	3	2	1	3	4	3	8	1	4	7	2	2	3	3	3	4

### SNP characterization of five Ethiopian strains

**Fig. 2.**
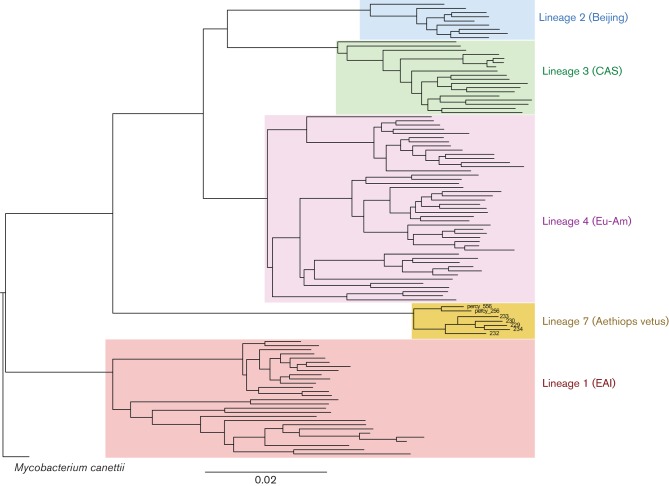
Parsimony-based phylogeny of Mtb using 78  613 concatenated variable positions (SNPs) found in at least one of 100 previously characterized RIVM strains of different lineages called against *M. canettii* CIPT 140070010, the genomically most distant strain among the *M. canettii* taxon (Supply *et al.*, 2013; Blouin *et al.*, 2014; Boritsch *et al.*, 2016). The scale bar represents 0.02 substitutions/site.

The five Ethiopian strains shared an average of 940 SNPs (range 888–986) and differed by an average of 183 SNPs (range 113–265) by pairwise comparisons. In the SNP-based phylogenetic tree, the five Ethiopian isolates clustered into one unique branch located between the ancient lineage 1 (East African Indian strains) and modern lineages 2, 3 and 4 [Beijing, Central Asian Strain (CAS) and Euro-American] (Fig. 2).

### LSP characterization of five Ethiopian strains by presence/absence of RDs and newly identified deletions

**Fig. 3.**
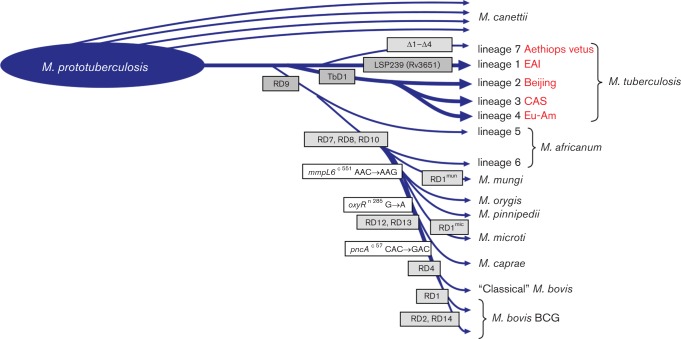
Phylogenetic lineage classification based on the presence or absence of regions of difference (RD) and smaller deletions.

All five Ethiopian strains contain all the genomic regions that are absent from *Mycobacterium bovis BCG Pasteur* (RD1 through RD9), with the exception of the RD3 region (corresponding to the prophage region PhiRv1) and RD11 (corresponding to the prophage region PhiRv2) (Fig. 3). The RD6 region was not tested as it contains Insertion Sequence (IS) elements. While absent in *M. tuberculosis* H37Rv, the TbD1 region, RvD1-RvD5 and RD239 (or LSP 239) as described by Brosch *et al.* (2002) and Gagneux *et al.* (2006) were conserved in the five Ethiopian strains. Fig. 3 visually summarizes these observations and shows the positioning of the five Ethiopian strains within the MTBC.

Four additional specific deletions (indicated as Δ1–Δ4 in Fig. 3) were found across all five strains: a 10 bp deletion in the *mmpl9* gene, a 27 bp deletion in the *lppH* gene (Fig. S1, available in the online Supplementary Material), a 1.3 kb fragment spanning genes *lppO*/*sseB* (Fig. S2), and a fragment of 3.3 kb spanning the genes *rv3467*/*rmlB2*/*mhpE* (Fig. S3). All four deletions were also confirmed in the lineage 7 Percy256 strain (Blouin *et al.*, 2012). Finally, in the region encompassing RD7 in Mtb H37Rv, the five Ethiopian strains present an extra 4.4 kb fragment that is absent from Mtb lineage 4 strains as well from all RD7-deleted MTBC members (*M. africanum* lineage 6, *Mycobacterium mungi*, *Mycobacterium orygis*, *M. bovis,* etc.) due to two independent deletion events (Fig. S4). However, this region seems to be present in all other tubercle bacilli (Fig. S4), including the *M. canettii* strains, in which the genes correspond to the orthologs of MCAN_19931, MCAN_19941, MCAN_19951 and MCAN_19961 (Supply *et al.*, 2013). Similarly, another genomic region of approximately 5 kb in the proximity of the *pknH* gene was present in the five Ethiopian strains and in *M. canettii.* This region is absent from Mtb H37Rv (Fig. S5) and many other Mtb strains of the modern lineages. An overview of what is known to date about the function of the genes/regions where these specific deletions take place is presented in Table S1. All additional smaller INDELs found amongst the five Ethiopian strains, mostly of one base pair, are listed in Table S2.

## Discussion

*Mycobacterium tuberculosis*, a member of the MTBC, has been a significant cause of morbidity and mortality among humans for thousands of years. Phylogenetic studies have shown that the MTBC comprises at least seven distinct lineages distributed in different geographical locations around the world. Current evidence indicates that the Horn of Africa is the most likely origin of MTBC from which it has spread to other parts of the world (Comas *et al.*, 2013).

Ethiopia is a country in the Horn of Africa with an estimated TB incidence of 224 cases/100 000 population (Global Tuberculosis Report, 2014). Molecular typing of isolates from various regions indicates that all major MTBC lineages are represented among TB patients in Ethiopia. A recent study conducted in the Amhara Region of Ethiopia identified a high proportion of ‘ Unknown’ spoligotype patterns (SIT910 and SIT1729) in addition to strains not previously described in the database (Yimer *et al.*, 2013). These strains have since been included in the recently described Mtb lineage 7.

The current study focused on the further characterization of five lineage 7 strains isolated in different parts of the Amhara Region in Ethiopia, the second most populous region in the country with an estimated 20 million inhabitants. One of the strains matched the SIT910 pattern of lineage 7 Percy556 from Djibouti (Blouin *et al.*, 2012), whilst another matched SIT1729. The results indicate that lineage 7 strains form a separate group as demonstrated by VNTR and SNP typing (Van Klingeren *et al.*, 2007). Both the analysis of RDs and SNPs in our study confirms the positioning of lineage 7 strains between ancient and modern lineages, as proposed by the SNP tree in Firdessa *et al.*(2013) and Comas* et al.* (2013). The inclusion of the phylogenetically most distant *M. canettii* strain STB-K (CIPT 1400700100) (Supply *et al.*, 2013; Blouin *et al.*, 2014; Boritsch *et al.*, 2016) as the outgroup has reinforced the clusters observed among the different Mtb.

We found four newly identified deletions characteristic of all five lineage 7 strains from the Amhara Region and the Percy256 strain. Our study also provides additional information on the genetic heterogeneity of Mtb lineage 7 strains. The strains in our dataset were found to be more heterogeneous (average of 183 SNPs ranging from 113 to 265) than the two Percy (256 and 556) strains described in Blouin *et al.* (2012) that differed by only eight SNPs. While Firdessa *et al.* (2013) identified lineage 7 strains in the Woldiya area, we showed that lineage 7 strains also circulate in other parts of the Amhara Region and among foreign-born patients from the Horn of Africa residing in The Netherlands.

There is a growing interest in conducting phylogeographic studies on MTBC isolates and it is likely that lineage 7 strains may be identified in other parts of the world. Mtb lineage 7 strains have previously been reported in Ethiopia (Firdessa *et al.*, 2013; Yimer *et al.*, 2013, 2015) and among Ethiopian immigrants in Djibouti (Blouin *et al.*, 2012). However, a specific designation that can distinguish these particular strains from other sub-lineages has not been proposed. It might therefore be opportune to designate this lineage ‘Aethiops vetus’. This name reflects the ancient origin of the new strains identified to date in Ethiopia and among Ethiopian immigrants abroad.

This geographical restriction is also in accordance with the low representation of lineage 7 strains in The Netherlands. Between 1993 and 2011 there have been 2455 TB cases in The Netherlands among foreign-born patients originating from the Horn of Africa; approximately a third of the strains belong to the East African Indian (EAI) lineage and the other two-thirds are either CAS or Euro-American.

Previous studies showed that the genomic diversity of MTBC has a significant impact on virulence and disease presentation among TB patients (Manca *et al.*, 1999; López-Granados *et al.*, 2003; Hernandez Pando *et al.*, 2010; Sreevatsan *et al.*, 1997). Further investigation of differences in virulence and immunogenicity among MTBC major lineages, including lineage 7 from Ethiopia and the Horn of Africa, is warranted to elucidate the pathogenesis, epidemiology and clinical consequences on TB disease. These studies will provide a better understanding of MTBC evolution and an indication of MTBC evolution under the current treatment regimens.

## Conclusion

The study confirms through different DNA typing techniques and whole-genome sequencing, the phylogenetic positioning of Mtb lineage 7 strains between the ancient lineage 1 and the TbD1-deleted, modern lineage 2, 3 and 4 strains. In addition, we have shown that this new lineage is not widespread in The Netherlands where the majority of TB patients are foreign-born. This may reflect that lineage 7 strains are not highly transmissible as compared to modern Mtb strains. To recognize the place of origin we propose to designate the lineage 7 strains as ‘Aethiops vetus*’.* Further studies are warranted to investigate the relevance of this special Mtb strain lineage with respect to clinical disease and transmission as compared to other members of MTBC in Ethiopia and the Horn of Africa.

## Supplementary Data

6882 Supplementary Data
