# Supplementary material for: Genomic characterization of Mycobacterium tuberculosis lineage 7 and a proposed name: ‘Aethiops vetus’
Source: Microb Genom. 2016 Jun 24;2(6):e000063. doi: 10.1099/mgen.0.000063 (PMC5320646; doi:10.1099/mgen.0.000063)
Supplement: Supplementary file 1 — Supplementary Data [file mgen-02-63-s001.pdf]

**Supplementary Material Legends**

**Fig. S1:** Identification of small genomic deletions in the genomes of Aethiops vetus strains. Panel A shows the BLAST comparison of the nucleotide sequence of gene mmpl9 from Aethiops vetus strain 262 used as query sequence in comparison to the orthologous sequence of M. tuberculosis H37Rv (sbjct). Panel B shows pairwise linear genomic comparisons of M. canettii, Aethiops vetus, M. tuberculosis H37Rv, and M. bovis AF2122/97. Red lines indicate collinear blocks of DNA-DNA similarity, whereas the white triangle indicates a specific 27bp deletion in the orthologous lppH region of M. tuberculosis lineage 7 Aethiops vetus.

**Fig. S2:** Identification of a specific 1.3 kb deletion in Aethiops vetus, concerning the orthologues of genes lppO and sseB. Panel A shows the linear genomic comparisons of M. canettii, M. tuberculosis strains Aethiops vetus, H37Rv, and M. bovis AF2122/97. Red lines indicate collinear blocks of DNA-DNA similarity, whereas the white triangles indicate the specific region absent from Aethiops vetus/lineage 7 strains. Panel B shows the BLASTN results obtained with the Aethiops vetus junction region of strain 262, used as query sequence, which confirms the specific 1.3 kb deletion relative to M. tuberculosis H37Rv (sbjct).

**Fig. S3:** Identification of a specific 3.3 kb deletion in M. tuberculosis Aethiops vetus strains concerning the orthologues of genes rv3467/rmlB2/mhpE. Panel A shows the linear genomic comparisons of M. canettii, M. tuberculosis Aethiops vetus, H37Rv, and M. bovis AF2122/97. Red lines indicate collinear blocks of DNA-DNA similarity, whereas the white triangles indicate the specific region absent from Aethiops vetus/lineage 7 strains. Panel B shows the BLASTN results obtained with the Aethiops vetus junction region of strain 262, used as query sequence, which confirms the specific 3,331 bp deletion relative to M. tuberculosis H37Rv (sbjct).

**Fig. S4:** Linear genomic comparison of M. canettii, M. tuberculosis Aethiops vetus, H37Rv, and M. bovis AF2122/97 sequences, covering the region of difference RD7. Red lines indicate collinear blocks of DNA-DNA similarity, whereas the white triangles indicate regions absent from selected strain lineages. The figure shows that in M. canettii and Aethiops vetus the genomic region encompassing orthologues of genes MCAN_19931-19961 are missing from M. tuberculosis H37Rv and other lineage 4 strains. An overlapping portion of this genomic region, located close to the terminus of replication region, was also subject of an independent genomic deletion (RD7) in the progenitor of M. africanum lineage 6 strains and animal strain lineages, including M. bovis.

**Fig.S5:** Linear genomic comparison of M. canettii, M. tuberculosis Aethiops vetus, H37Rv, and M. bovis AF2122/97 sequences, covering the region encoding for serine threonine kinase PknH. Red lines indicate collinear blocks of DNA-DNA similarity, whereas the white triangles indicate regions absent from M. tuberculosis H37Rv due to recombination of two pknH copies and deletion of the adjacent gene (orthologue of MCAN_12811). Interestingly, the full pknH region was also described as being present in M. africanum, which argues that the recombined region in M. bovis is due to an independent recombination event in the M. africanum-M. bovis lineage, which is also suggested by the sequence differences in the pknH gene between M. tuberculosis H37Rv and M. bovis, (as indicated by a white block of distant similarity).

**Table S1:** Additional smaller INDELs found amongst the 5 M. tuberculosis lineage 7/Aethiops vetus strains (against reference strain H37Rv).

**Table S2:** Overview of relevant functional studies on genes/regions where LSPs/deletions specific to lineage 7 Aethiops vetus occur.

Supplementary Figure 1

**A**

```
Query: 2281    gacattcgagacggtaccaagtacgatatcctcatcgttggtatagccgcggtatgcctg 2340
               ||||||||||||||||||||||||||||||||||||||||||||||||||||||||||||
Sbjct: 2616973 gacattcgagacggtaccaagtacgatatcctcatcgttggtatagccgcggtatgcctg 2617032

Query: 2341    gtatttattgtcatgctcatgattacccagagcctgattgcgtcactcgtcattgttggc 2400
               ||||||||||||||||||||||||||||||||||||||||||||||||||||||||||||
Sbjct: 2617033 gtatttattgtcatgctcatgattacccagagcctgattgcgtcactcgtcattgttggc 2617092

Query: 2401    acggtacttctgtcattgggtactgcgttcggactgtccgtgctcat----------ttt 2450
               |||||||||||||||||||||||||||||||||||||||||||||||          |||
Sbjct: 2617093 acggtacttctgtcattgggtactgcgttcggactgtccgtgctcatctggcagcacttt 2617152

Query: 2451    gtcggtctccaggtgcattggacgatcgtcgcgatgtctgtcatcgtcttgctggccgtc 2510
               ||||||||||||||||||||||||||||||||||||||||||||||||||||||||||||
Sbjct: 2617153 gtcggtctccaggtgcattggacgatcgtcgcgatgtctgtcatcgtcttgctggccgtc 2617212

Query: 2511    ggttctgactacaacctccttttggtgtcccggttcaaggaggaggtcggcgctggatta 2570
               ||||||||||||||||||||||||||||||||||||||||||||||||||||||||||||
Sbjct: 2617213 ggttctgactacaacctccttttggtgtcccggttcaaggaggaggtcggcgctggatta 2617272
```

**B**

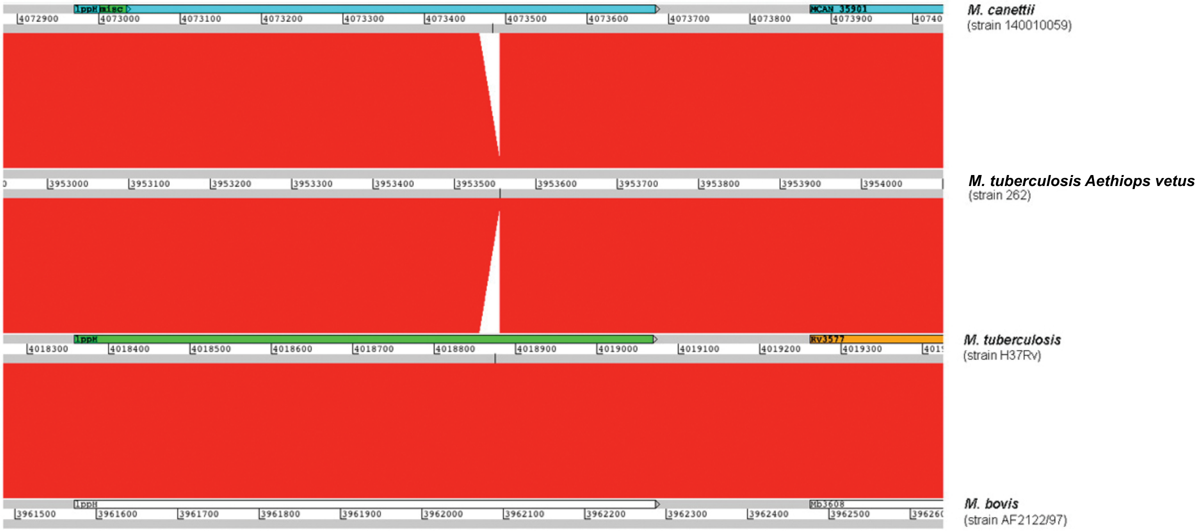

Supplementary Figure 2

**A**

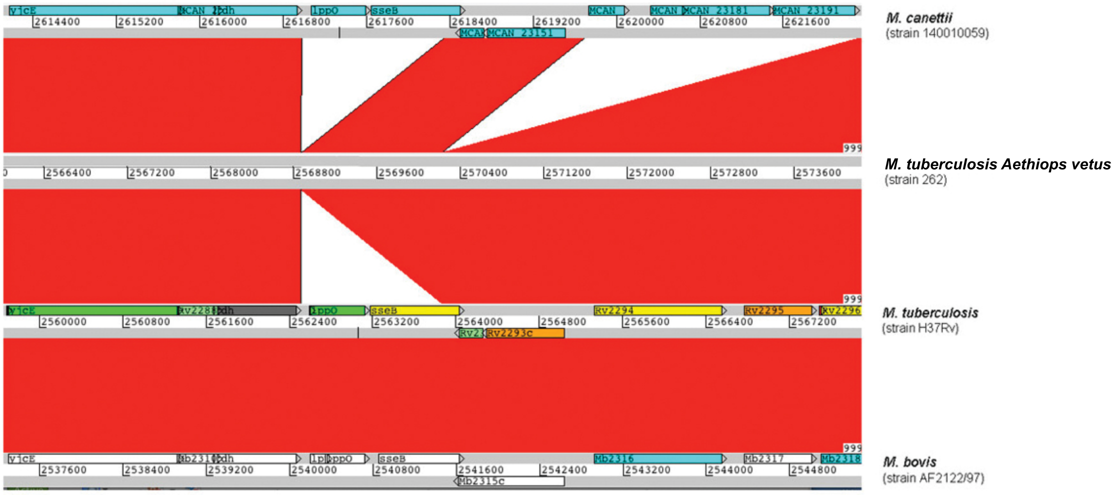


**B**

```
Query: 1       cgaatccaggaacctgttccgggacatctacctgaaggtaaccgctgacggcaagaaaat 60
               ||||||||||||||||||||||||||||||||||||||||||||||||||||||||||||
Sbjct: 2562265 cgaatccaggaacctgttccgggacatctacctgaaggtaaccgctgacggcaagaaaat 2562324

Query: 61      ggaaaatgcatcgattgcggttgccaacgtagcgcaggaccaattcctgctgctcttggc 120
               ||||||||||||||||||||||||||||||||||||||||||||||||||||||||||||
Sbjct: 2562325 ggaaaatgcatcgattgcggttgccaacgtagcgcaggaccaattcctgctgctcttggc 2562384

Query: 121     agagggaactgaggaccagcccgttgcagccgagactctccaagaccacgactgctccat 180
               ||||||||||||||||||||||||||||||||||||||||||||||||||||||||||||
Sbjct: 2562385 agagggaactgaggaccagcccgttgcagccgagactctccaagaccacgactgctccat 2562444

Query: 181     caccaagtcctgatagcacgatgccagcgggccacacgacagggcgcagtgtgcgaacct 240
               ||||||||||||||||||||||||||||||||||||||||||||||||||||||||||||
Sbjct: 2562445 caccaagtcctgatagcacgatgccagcgggccacacgacagggcgcagtgtgcgaacct 2562504

Query: 241     gaccccgcca 250
               ||||||||||
Sbjct: 2562505 gaccccgcca 2562514


 Score =  329 bits (166), Expect = 1e-90
 Identities = 169/170 (99%)
 Strand = Plus / Plus

Query: 251     tggccgcgtgggtgtctactgcggctcgggtgtcagcgcagctgtcatcgtcgcggcact 310
               ||||||||||||||||||||||||||||||||||||||||||||||||||||||||||||
Sbjct: 2563875 tggccgcgtgggtgtctactgcggctcgggtgtcagcgcagctgtcatcgtcgcggcact 2563934

Query: 311     ggcagtgatcggccaggatgcggcgctgtttccagggtcatggtcggagtggagttcgga 370
               ||||||||||||||||||||||| ||||||||||||||||||||||||||||||||||||
Sbjct: 2563935 ggcagtgatcggccaggatgcggagctgtttccagggtcatggtcggagtggagttcgga 2563994

Query: 371     tccgacccgtcccgtcggccgtggcactgcatagtcagacgccggcccag 420
               ||||||||||||||||||||||||||||||||||||||||||||||||||
Sbjct: 2563995 tccgacccgtcccgtcggccgtggcactgcatagtcagacgccggcccag 2564044
```

Supplementary Figure 3

**A**

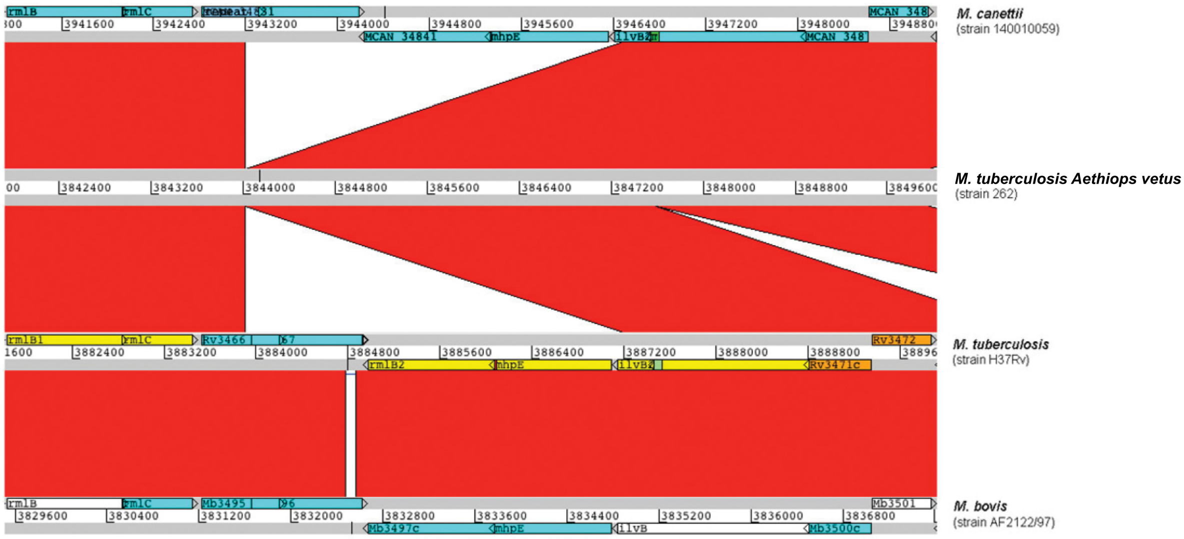


**B**

```
Query: 1        tgggtcacgcgttgatcaaccaacttgacgcccaagccagcgaggaagaactgggcggca 60
                ||||||||||||||||||||||||||||||||||||||||||||||||||||||||||||
Sbjct: 3883682  tgggtcacgcgttgatcaaccaacttgacgcccaagccagcgaggaagaactgggcggca 3883741


Query: 61       cgctgtgctgcgcgctggccaaccggttacgcatcaccaagcccgacgccgccctacgca 120
                |||||||||||||||||||||||||||||||||||||||||||||||||||||   ||||
Sbjct: 3883742  cgctgtgctgcgcgctggccaaccggttacgcatcaccaagcccgacgccgcccggcgca 3883801


Query: 121      tcgccgacgccgccgatctcggacctcgtcgagcactcaccggcgaaccgctagccccac 180
                |||||||||||||||||||||||||||||||||||||||||| |||||||||||||||||
Sbjct: 3883802  tcgccgacgccgccgatctcggacctcgtcgagcactcaccggtgaaccgctagccccac 3883861


Query: 181      agttgaccgccaccgccaccgcccaacgccagggcctgatcggcga 226
                ||||||||||||||||||||||||||||||||||||||||||||||
Sbjct: 3883862  agttgaccgccaccgccaccgcccaacgccagggcctgatcggcga 3883907



 Score =  289 bits (146), Expect = 8e-79
 Identities = 146/146 (100%)
 Strand = Plus / Plus


Query: 225      gacgcagggaccggtggcggccaacgcttttttgcacaccggcgcgcagctcgcagcgcgt 284
                ||||||||||||||||||||||||||||||||||||||||||||||||||||||||||||
Sbjct: 3887201  gacgcagggaccggtggcggccaacgcttttttgcacaccggcgcgcagctcgcagcgcgt 3887260


Query: 285      atcgacccgaatcccttccgctccaagggcgcgggccatcgccgccagatcgttcgcgcc 344
                ||||||||||||||||||||||||||||||||||||||||||||||||||||||||||||
Sbjct: 3887261  atcgacccgaatcccttccgctccaagggcgcgggccatcgccgccagatcgttcgcgcc 3887320


Query: 345      gatgcgagcgaccggcgacggatcca 370
                ||||||||||||||||||||||||||
Sbjct: 3887321  gatgcgagcgaccggcgacggatcca 3887346
```

Supplementary Figure 4

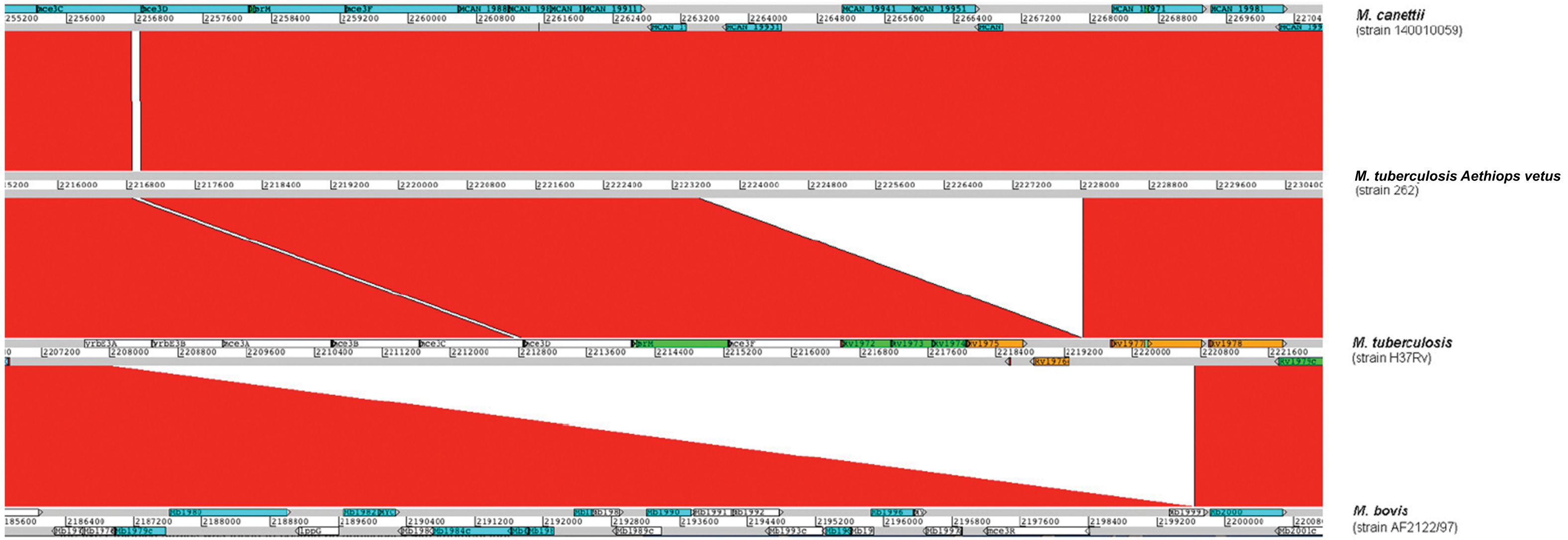

Supplementary Figure 5

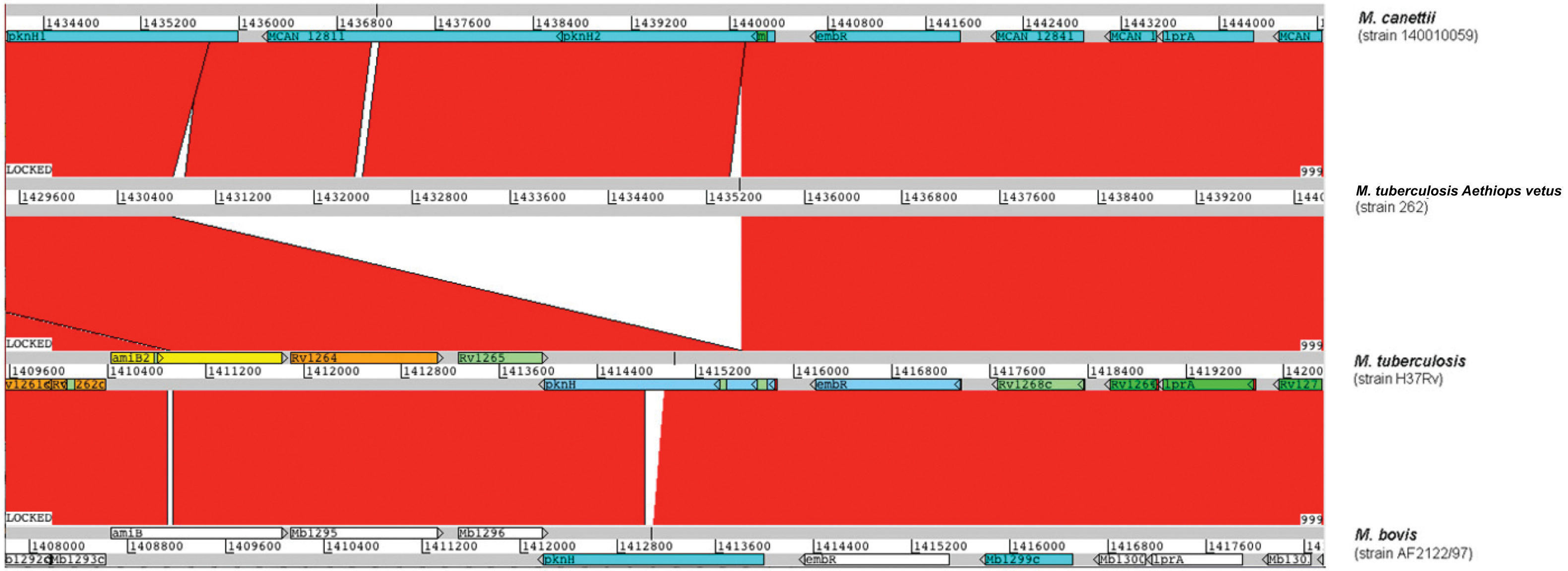

Supplementary Table 1

| Gene name | Rv number | Functional category | Function | Relevant findings |
| --- | --- | --- | --- | --- |
| mmpL9 | Rv2339 | Cell wall and cell processes | Unknown. Thought to be involved in fatty acid transport. | • Decrease in survival observed in mmpL9 mutant in hydrogen peroxide (Mestre O et al., 2013).<br>• MmpL-mediated lipid secretion contributes to innate ability of pathogen to survive intracellularly and contributes directly to host-pathogen dialogue that determines the ultimate outcome of infection (Domenech P., 2004). |
| lppH | Rv3576 | Cell wall and cell processes | Unknown. | |
| lppO | Rv2290 | Cell wall and cell processes | Unknown. | |
| sseB | Rv2291 | Intermediary metabolism and respiration | Unknown. | • Has been implicated in pathogenesis (Graham, JE et al., 1999 & McKinney, JD et al., 2000). |
| Rv3467 | Rv3467 | insertion seqs and phages | Unknown. | |
| galE1 (rmlB2) | Rv3634c | Intermediary metabolism and respiration | Involved in galactofuranosyl biosynthesis. | • rmlB2 genes observed to be upregulated in macrophages (Fontan, P., 2008), thus a putative bacterial pathogenic factor relevant for the intracellular survival of MTB. |
| mhpE | Rv3469c | Intermediary metabolism and respiration | Involved in aromatic hydrocarbons degradation | |
| pknH | Rv1266c | Regulatory proteins | Involved in signal transduction (via phosphorylation). | • Its disruption in H37Rv confers a higher bacillary load (hypervirulence) during the chronic phase of infection in BALB/c mice (Papavinasasasundaram, KG et al., 2005).<br>• Its differential expression in response to stress conditions indicate its ability to regulate cellular events promoting bacterial adaptation to environmental change (Sharma, K., et al., 2004). |

Supplementary Table 2

| position | mutation | annotation | gene | description |
|---|---|---|---|---|
| 30,980 | +CGAT | coding (1259/1347 nt) | Rv0026 → | hypothetical protein |
| 125,830 | +A | coding (4712/4899 nt) | ctpI ← | cation-transporter ATPase I |
| 131,174 | +G | intergenic (-70/-208) | Rv0108c ← / → PE_PGRS1 | hypothetical protein/PE-PGRS family protein |
| 139,556 | +C | coding (1044/1161 nt) | hddA → | D-alpha-D-heptose-7-phosphate kinase |
| 194,305 | +GG | coding (634/795 nt) | Rv0165c ← | GntR family transcriptional regulator |
| 230,576 | +T | intergenic (+114/-323) | Rv0194 → / → Rv0195 | drugs-transport transmembrane ATP-binding protein ABC transporter/two component transcriptional regulatory protein |
| 234,496 | +GT | coding (2266/2289 nt) | Rv0197 → | oxidoreductase |
| 289,125 | +T | coding (22/234 nt) | Rv0239 → | hypothetical protein |
| 293,628 | +C | intergenic (+135/+170) | fadA2 → / ← fadE5 | acetyl-CoA acetyltransferase/acyl-CoA dehydrogenase FADE5 |
| 424,320 | +C | coding (375/426 nt) | PPE7 ← | PPE family protein |
| 424,790 | +TA | coding (9890/9903 nt) | PPE8 ← | PPE family protein |
| 467,497 | +G | coding (505/543 nt) | PPE9 ← | PPE family protein |
| 467,508 | +G | coding (494/543 nt) | PPE9 ← | PPE family protein |
| 592,759 | +A | coding (1106/1131 nt) | galE2 → | UDP-glucose 4-epimerase |
| 641,719 | +C | coding (1093/1716 nt) | fadD8 ← | acyl-CoA synthetase |
| 688,792 | +G | coding (761/828 nt) | mce2B → | MCE-family protein MCE2B |
| 750,089 | +C | coding (90/1506 nt) | Rv0654 → | dioxygenase |
| 874,835 | +CG | coding (603/711 nt) | ptrBa → | oligopeptidase B |
| 976,897 | +G | coding (1307/1332 nt) | PPE13 ← | PPE family protein |
| 1,010,204 | +G | coding (69/1599 nt) | Rv0907 → | hypothetical protein |
| 1,086,490 | +C | coding (856/1590 nt) | accD2 ← | acetyl-/propionyl-CoA carboxylase subunit beta |
| 1,090,188 | +G | intergenic (-13/-185) | Rv0976c ← / → PE_PGRS16 | hypothetical protein/PE-PGRS family protein |
| 1,131,229 | +G | coding (102/294 nt) | Rv1012 → | hypothetical protein |
| 1,165,521 | +A | intergenic (-22/+260) | Rv1042c ← / ← Rv1043c | IS like-2 transposase/hypothetical protein |
| 1,168,715 | +T | coding (514/525 nt) | Rv1046c ← | hypothetical protein |
| 1,273,250 | +A | coding (828/912 nt) | mmpL13a → | transmembrane transport protein MmpL13A |
| 1,365,837 | +GG | intergenic (+29/-38) | Rv1222 → / → htrA | hypothetical protein/serine protease HtrA |
| 1,474,959 | +CA | noncoding (1302/3138 nt) | rrl → | ribosomal RNA 23S |
| 1,488,557 | +C | coding (1409/1812 nt) | PE_PGRS24 ← | PE-PGRS family protein |
| 1,541,066 | +C | coding (47/786 nt) | lprF → | lipoprotein LprF |
| 1,696,499 | +CGGT | intergenic (-56/+228) | Rv1506c ← / ← Rv1507c | hypothetical protein/hypothetical protein |
| 1,753,519 | +C | coding (10/528 nt) | fadD11.1 → | fatty-acid-CoA ligase |
| 1,878,817 | +G | coding (3514/6381 nt) | pks7 → | polyketide synthase pks7 |
| 1,894,301 | +10 bp | coding (1042/1119 nt) | Rv1668c ← | macrolide-transport ATP-binding protein ABC transporter |
| 2,001,789 | +GG | coding (1176/1857 nt) | PE_PGRS31 → | PE-PGRS family protein |
| 2,090,401 | +12 bp | coding (318/1038 nt) | Rv1841c ← | hypothetical protein |
| 2,109,523 | +G | intergenic (+53/-21) | Rv1861 → / → adhA | transmembrane protein/alcohol dehydrogenase AdhA |
| 2,133,474 | +15 bp | coding (219/462 nt) | Rv1883c ← | hypothetical protein |
| 2,137,522 | +10 bp | coding (558/561 nt) | Rv1888c ← | transmembrane protein |
| 2,143,523 | +T | coding (1003/1155 nt) | Rv1895 → | dehydrogenase |
| 2,161,343 | +T | coding (881/1104 nt) | aceAa → | isocitrate lyase |
| 2,207,591 | +C | intergenic (-789/-109) | mce3R ← / → yrbE3A | TetR family transcriptional regulator/integral membrane protein YrbE3A |
| 2,218,577 | +C | coding (526/666 nt) | Rv1975 → | hypothetical protein |
| 2,342,650 | +11 bp | coding (843/1137 nt) | Rv2084 → | hypothetical protein |
| 2,358,029 | +G | intergenic (-1352/-360) | Rv2097c ← / → Rv2100 | hypothetical protein/hypothetical protein |
| 2,421,975 | +GGAA | coding (304/636 nt) | Rv2160A ← | hypothetical protein |
| 2,523,205 | +CGC | intergenic (+30/+36) | Rv2248 → / ← glpD1 | hypothetical protein/glycerol-3-phosphate dehydrogenase |
| 2,536,625 | +G | coding (1726/1779 nt) | Rv2264c ← | hypothetical protein |
| 2,564,368 | +C | coding (665/741 nt) | Rv2293c ← | hypothetical protein |
| 2,606,797 | +C | coding (1525/1614 nt) | Rv2333c ← | integral membrane transport protein |
| 2,632,341 | +A | intergenic (-266/+582) | plcA ← / ← PPE38 | membrane-associated phospholipase C/PPE family protein |
| 2,704,885 | +21 bp | coding (189/822 nt) | Rv2407 → | ribonuclease Z |
| 2,960,944 | +C | coding (1498/2337 nt) | PE_PGRS46 ← | PE-PGRS family protein |
| 3,131,472 | +9 bp | coding (302/2430 nt) | Rv2823c ← | hypothetical protein |
| 3,194,241 | +G | coding (1308/1383 nt) | Rv2885c ← | transposase |
| 3,293,700 | +G | coding (2654/4851 nt) | pks1 ← | polyketide synthase PKS1 |
| 3,473,996 | +A | intergenic (-92/-11) | prfB ← / → fprA | peptide chain release factor 2/NADPH:adrenodoxin oxidoreductase FPRA (NADPH-ferredoxin reductase) |
| 3,539,139 | +G | coding (635/1347 nt) | aofH → | flavin-containing monoamine oxidase |
| 3,590,686 | +C | intergenic (+69/+6) | Rv3212 → / ← Rv3213c | hypothetical protein/SOJ/PARA-like protein |
| 3,610,391 | +C | coding (799/816 nt) | Rv3234c ← | hypothetical protein |
| 3,723,901 | +T | coding (246/387 nt) | Rv3337 → | hypothetical protein |
| 3,794,867 | +CA | coding (1/1611 nt) | dxs2 ← | 1-deoxy-D-xylulose-5-phosphate synthase |
| 3,801,551 | +T | intergenic (+88/-102) | Rv3387 → / → PE_PGRS52 | transposase/PE-PGRS family protein |
| 3,819,797 | +CA | coding (1756/2361 nt) | Rv3401 → | hypothetical protein |
| 4,170,964 | +A | coding (751/930 nt) | Rv3725 → | oxidoreductase |
| 4,197,138 | +T | intergenic (+31/-98) | Rv3747 → / → Rv3748 | hypothetical protein/hypothetical protein |
| 4,277,517 | +G | coding (947/1515 nt) | PE_PGRS62 → | PE-PGRS family protein |
| 4,306,873 | +A | coding (7/792 nt) | Rv3833 → | AraC family transcriptional regulator |
| 4,337,820 | +G | coding (1044/1173 nt) | Rv3860 → | hypothetical protein |
| 4,341,396 | +11 bp | coding (1127/1209 nt) | Rv3864 → | hypothetical protein |
| 4,358,979 | +G | coding (804/2190 nt) | Rv3879c ← | hypothetical protein |
| 4,360,680 | +A | coding (1246/1383 nt) | Rv3881c ← | hypothetical protein |
| 4,383,144 | +CGGGG | coding (497/633 nt) | Rv3897c ← | hypothetical protein |
| 34,511 | Δ1 bp | coding (217/2316 nt) | bioF2 → | 8-amino-7-oxononanoate synthase BioF2 |
| 77,818 | Δ1 bp | coding (1079/1278 nt) | glyA ← | serine hydroxymethyltransferase |
| 105,175 | Δ1 bp | coding (41/411 nt) | Rv0095c ← | hypothetical protein |
| 137,851 | Δ1 bp | coding (533/591 nt) | gmhA → | phosphoheptose isomerase |
| 154,179 | Δ53 bp | between 53 bp Mycobacterial Interspersed Repetitive Unit, Class II | treS → / → Rv0127 | trehalose synthase TRES/hypothetical protein |
| 173,080 | Δ1 bp | coding (870/933 nt) | Rv0146 → | hypothetical protein |
| 208,317 | Δ1 bp | coding (866/969 nt) | Rv0176 → | mce associated transmembrane protein |
| 289,070 | Δ1 bp | intergenic (+28/-34) | Rv0238 → / → Rv0239 | TetR family transcriptional regulator/hypothetical protein |
| 323,029 | Δ1 bp | coding (245/510 nt) | Rv0268c ← | hypothetical protein |
| 364,499 | Δ1 bp | intergenic (+30/-106) | Rv0301 → / → Rv0302 | hypothetical protein/TetR/ACRR family transcriptional regulator |
| 373,283 | Δ1 bp | coding (2429/2892 nt) | PPE6 ← | PPE family protein |
| 374,762 | Δ15 bp | coding (936-950/2892 nt) | PPE6 ← | PPE family protein |
| 384,541 | Δ1 bp | coding (7/615 nt) | Rv0316 → | muconolactone isomerase |
| 428,084 | Δ1 bp | coding (6596/9903 nt) | PPE8 ← | PPE family protein |
| 479,982 | Δ1 bp | coding (194/372 nt) | Rv0401 → | transmembrane protein |
| 597,146 | Δ1 bp | coding (388/444 nt) | mmpS2 → | membrane protein |
| 843,931 | Δ1 bp | coding (484/1173 nt) | fadE9 ← | acyl-CoA dehydrogenase FADE9 |
| 854,253 | Δ1 bp | intergenic (-27/+14) | Rv0759c ← / ← Rv0760c | hypothetical protein/hypothetical protein |
| 925,343 | Δ1 bp | coding (393/414 nt) | PE_PGRS12 → | PE-PGRS family protein |
| 952,811 | Δ1 bp | intergenic (+100/-14) | far → / → Rv0856 | fatty-acid-CoA racemase/hypothetical protein |
| 1,218,649 | Δ9 bp | coding (2181-2189/2562 nt) | PE_PGRS22 → | PE-PGRS family protein |
| 1,340,653 | Δ1 bp | intergenic (+129/-6) | PPE18 → / → esxK | PPE family protein/Esat-6 like protein esxK (Esat-6 like protein 3) |
| 1,385,074 | Δ3 bp | coding (1602-1604/1689 nt) | PE_PGRS23 ← | PE-PGRS family protein |
| 1,415,281 | Δ13 bp | coding (548-560/1881 nt) | pknH ← | Ser/Thr protein kinase H |
| 1,502,753 | Δ9 bp | coding (113-121/441 nt) | Rv1334 → | hypothetical protein |
| 1,546,822 | Δ1 bp | coding (811/981 nt) | Rv1373 → | glycolipid sulfotransferase |
| 1,561,664 | Δ1 bp | coding (201/309 nt) | PE15 → | PE family protein |
| 1,655,680 | Δ9 bp | coding (1034-1042/1113 nt) | PE_PGRS29 ← | PE-PGRS family protein |
| 1,677,116 | Δ3 bp | coding (176-178/435 nt) | Rv1487 → | hypothetical protein |
| 1,714,132 | Δ1 bp | intergenic (+79/+40) | fadD25 → / ← mmpL12 | acyl-CoA synthetase/transmembrane transport protein MmpL12 |
| 1,972,840 | Δ1 bp | coding (703/1431 nt) | pknF → | anchored-membrane serine/threonine-protein kinase PKNF (protein kinase F) (STPK F) |
| 1,978,239 | Δ2 bp | coding (1328-1329/1599 nt) | fadD1 ← | acyl-CoA synthetase |
| 1,992,324 | Δ1 bp | coding (254/2745 nt) | wag22 ← | PE-PGRS family protein |
| 2,058,119 | Δ1 bp | coding (592/666 nt) | Rv1815 → | hypothetical protein |
| 2,094,912 | Δ6 bp | coding (272-277/1458 nt) | gnd1 ← | 6-phosphogluconate dehydrogenase |
| 2,127,928 | Δ3 bp | coding (2025-2027/2064 nt) | Rv1877 → | integral membrane protein |
| 2,183,462 | Δ1 bp | coding (91/498 nt) | tpx → | thiol peroxidase |
| 2,189,574 | Δ3 bp | coding (1079-1081/2520 nt) | Rv1937 → | oxygenase |
| 2,192,436 | Δ1 bp | coding (343/516 nt) | Rv1939 → | oxidoreductase |
| 2,214,655 | Δ9 bp | coding (533-541/1134 nt) | lprM → | MCE-family lipoprotein LprM |
| 2,239,633 | Δ1 bp | coding (630/954 nt) | Rv1996 → | hypothetical protein |
| 2,339,611 | Δ6 bp | coding (903-908/2166 nt) | Rv2082 → | hypothetical protein |
| 2,357,269 | Δ3 bp | intergenic (-592/-1118) | Rv2097c ← / → Rv2100 | hypothetical protein/hypothetical protein |
| 2,368,565 | Δ1 bp | intergenic (+123/+418) | PPE36 → / ← prcA | PPE family protein/proteasome (alpha subunit) PrcA |
| 2,387,186 | Δ5 bp | intergenic (+15/+12) | Rv2125 → / ← PE_PGRS37 | hypothetical protein/PE-PGRS family protein |
| 2,448,884 | Δ2 bp | coding (725-726/1803 nt) | fadD15 → | long-chain-fatty-acid-CoA ligase fadD15 (fatty-acid-CoA synthetase) (fatty-acid-CoA synthase) |
| 2,453,896 | Δ1 bp | coding (78/1938 nt) | Rv2191 → | hypothetical protein |
| 2,525,723 | Δ1 bp | coding (322/420 nt) | Rv2250A → | flavoprotein |
| 2,534,563 | Δ2 bp | coding (989-990/1083 nt) | Rv2262c ← | hypothetical protein |
| 2,684,690 | Δ1 bp | coding (12/1692 nt) | nirA → | ferredoxin-dependent nitrite reductase NIRA |
| 2,724,202 | Δ20 bp | intergenic (-19/+9) | Rv2426c ← / ← proA | hypothetical protein/gamma-glutamyl phosphate reductase |
| 2,798,719 | Δ1 bp | coding (2162/3414 nt) | Rv2488c ← | LuxR family transcriptional regulator |
| 2,803,090 | Δ18 bp | coding (3130-3147/4983 nt) | PE_PGRS43 ← | PE-PGRS family protein |
| 2,805,386 | Δ1 bp | coding (851/4983 nt) | PE_PGRS43 ← | PE-PGRS family protein |
| 2,881,598 | Δ1 bp | coding (190/294 nt) | Rv2561 → | hypothetical protein |
| 2,957,569 | Δ1 bp | intergenic (+137/-3) | Rv2630 → / → Rv2631 | hypothetical protein/hypothetical protein |
| 3,020,370 | Δ9 bp | coding (80-88/258 nt) | Rv2706c ← | hypothetical protein |
| 3,190,146 | Δ1 bp | coding (7/570 nt) | Rv2879c ← | hypothetical protein |
| 3,331,362 | Δ2 bp | coding (250-251/255 nt) | Rv2975c ← | hypothetical protein |
| 3,381,237 | Δ1 bp | intergenic (-244/+138) | PE29 ← / ← Rv3023c | PE family protein/transposase |
| 3,415,181 | Δ14 bp | intergenic (-223/+241) | nrdH ← / ← Rv3054c | glutaredoxin electron transport protein NrdH/hypothetical protein |
| 3,426,069 | Δ6 bp | coding (486-491/1524 nt) | ligB → | ATP-dependent DNA ligase |
| 3,580,637 | Δ1 bp | coding (112/159 nt) | Rv4008 → | hypothetical protein |
| 3,627,387 | Δ1 bp | intergenic (-38/+32) | mtrA ← / ← tmk | two component sensory transduction transcriptional regulatory protein MTRA/thymidylate kinase |
| 3,683,269 | Δ1 bp | coding (2695/2913 nt) | atsB ← | arylsulfatase AtsB |
| 3,734,179 | Δ1 bp | coding (2757/7572 nt) | PPE54 ← | PPE family protein |
| 3,742,992 | Δ1 bp | intergenic (-218/+206) | PE_PGRS50 ← / ← Rv3346c | PE-PGRS family protein/hypothetical protein |
| 3,827,445 | Δ3 bp | coding (1281-1283/1737 nt) | choD ← | cholesterol oxidase precursor |
| 3,844,757 | Δ1 bp | coding (1214/1233 nt) | Rv3428c ← | transposase |
| 3,862,473 | Δ1 bp | intergenic (-83/+151) | rplM ← / ← esxT | 50S ribosomal protein L13/ESAT-6 like protein ESXT |
| 3,874,723 | Δ1 bp | coding (320/333 nt) | Rv3453 → | transmembrane protein |
| 3,890,100 | Δ1 bp | coding (634/786 nt) | bpoA ← | peroxidase BpoA |
| 4,035,996 | Δ1 bp | coding (140/828 nt) | Rv3594 → | hypothetical protein |
| 4,095,002 | Δ1 bp | coding (299/378 nt) | Rv3655c ← | hypothetical protein |
| 4,095,297 | Δ1 bp | coding (4/378 nt) | Rv3655c ← | hypothetical protein |
| 4,169,667 | Δ1 bp | coding (201/243 nt) | cut5a → | cutinase precursor |
| 4,189,684 | Δ12 bp | coding (538-549/948 nt) | PPE66 ← | PPE family protein |
| 4,198,612 | Δ1 bp | intergenic (-15/-262) | Rv3750c ← / → Rv3751 | excisionase/integrase |
| 4,307,622 | Δ9 bp | coding (756-764/792 nt) | Rv3833 → | AraC family transcriptional regulator |
| 4,338,596 | Δ1 bp | intergenic (-75/-253) | whiB6 ← / → Rv3863 | transcriptional regulatory protein WHIB-like WHIB6/hypothetical protein |
| 4,359,229 | Δ1 bp | coding (554/2190 nt) | Rv3879c ← | hypothetical protein |
| 4,386,640 | Δ9 bp | coding (167-175/450 nt) | Rv3901c ← | hypothetical protein |
| 4,400,661 | Δ1 bp | coding (476/669 nt) | sigM → | RNA polymerase sigma factor SigM |
| 3,925,504 | Δ5 bp | coding (615-619/1509 nt) | fadD17 → | acyl-CoA synthetase |
| 2,133,348 | +G | coding (345/462 nt) | Rv1883c ← | hypothetical protein |
| 2,881,582 | +T | coding (174/294 nt) | Rv2561 → | hypothetical protein |
| 3,111,343 | +C | coding (481/1044 nt) | Rv2802c ← | hypothetical protein |
| 1,026,917 | Δ1 bp | intergenic (-101/-187) | Rv0920c ← / → Rv0921 | transposase/resolvase |
| 2,469,918 | Δ1 bp | coding (546/1077 nt) | Rv2205c ← | hypothetical protein |
| 3,120,177 | Δ144 bp | between 36 bp direct repeat | Rv2813 → / ← Rv2814c | hypothetical protein/transposase |
| 4,375,354 | Δ1 bp | coding (330/1200 nt) | PPE69 ← | PPE family protein |
| 1,002,282 | +CC | coding (134/1608 nt) | Rv0897c ← | oxidoreductase |
| 1,103,437 | +A | coding (896/2568 nt) | Rv0987 → | adhesion component transport transmembrane protein ABC transporter |
| 2,566,766 | +G | intergenic (+216/-6) | Rv2294 → / → Rv2295 | aminotransferase/hypothetical protein |
| 2,782,084 | +27 bp | coding (179/4875 nt) | gdh ← | NAD-dependent glutamate dehydrogenase |
| 3,907,771 | +T | coding (105/324 nt) | Rv3488 → | hypothetical protein |
| 4,359,186 | +54 bp | coding (597/2190 nt) | Rv3879c ← | hypothetical protein |
| 737,747 | Δ5 bp | coding (399-403/882 nt) | mmaA3 ← | methoxy mycolic acid synthase |
| 943,981 | Δ1 bp | coding (214/1515 nt) | Rv0846c ← | oxidase |
| 1,543,532 | Δ1 bp | coding (174/1470 nt) | Rv1371 → | hypothetical protein |
| 1,691,287 | Δ1 bp | coding (438/1029 nt) | Rv1500 → | glycosyltransferase |
| 1,820,293 | Δ1 bp | coding (1401/1731 nt) | cydC ← | cytochrome' transport transmembrane ATP-binding protein ABC transporter CydC |
| 3,378,653 | Δ1 bp | intergenic (-410/+58) | PPE46 ← / ← esxR | PPE family protein/secreted ESAT-6 like protein ESXR (TB10.3) (ESAT-6 like protein 9) |
| 3,907,467 | Δ7 bp | intergenic (-289/-194) | lipF ← / → Rv3488 | esterase/lipase LipF/hypothetical protein |
| 4,031,612 | Δ268 bp | coding (1280-1547/1755 nt) | PE_PGRS58 ← | PE-PGRS family protein |
| 4,032,430 | Δ3 bp | coding (727-729/1755 nt) | PE_PGRS58 ← | PE-PGRS family protein |
| 4,081,108 | Δ1 bp | intergenic (-343/-52) | Rv3642c ← / → Rv3643 | hypothetical protein/hypothetical protein |
| 4,387,690 | Δ12 bp | coding (195-206/531 nt) | Rv3902c ← | hypothetical protein |
| 357,261 | +C | coding (804/1203 nt) | Rv0293c ← | hypothetical protein |
| 3,966,640 | +G | coding (399/1155 nt) | Rv3529c ← | hypothetical protein |
| 791,029 | Δ1 bp | coding (45/1050 nt) | Rv0690c ← | hypothetical protein |
| 1,338,795 | Δ3 bp | intergenic (-282/-206) | Rv1194c ← / → PE13 | hypothetical protein/PE family protein |
| 1,541,079 | Δ1 bp | coding (60/786 nt) | lprF → | lipoprotein LprF |
| 37,927 | Δ1 bp | coding (669/1689 nt) | fadD34 → | fatty-acid-CoA ligase |
| 691,888 | Δ1 bp | coding (1388/1527 nt) | mce2D → | MCE-family protein MCE2D |
| 2,960,896 | Δ18 bp | coding (1529-1546/2337 nt) | PE_PGRS46 ← | PE-PGRS family protein |
| 3,119,884 | Δ147 bp | between 36 bp direct repeat | Rv2813 → / ← Rv2814c | hypothetical protein/transposase |
| 3,687,568 | Δ1 bp | coding (10/666 nt) | phoY1 ← | phosphate transporter PhoU |
| 3,734,936 | Δ1 bp | coding (2000/7572 nt) | PPE54 ← | PPE family protein |
| 3,905,040 | Δ3 bp | coding (525-527/945 nt) | Rv3485c ← | short-chain dehydrogenase |
